# Common genetic variation in the Estrogen Receptor Beta (ESR2) gene and osteoarthritis: results of a meta-analysis

**DOI:** 10.1186/1471-2350-11-164

**Published:** 2010-11-16

**Authors:** Hanneke JM Kerkhof, Ingrid Meulenbelt, Andrew Carr, Antonio Gonzalez, Deborah Hart, Albert Hofman, Margreet Kloppenburg, Nancy E Lane, John Loughlin, Michael C Nevitt, Huibert AP Pols, Fernando Rivadeneira, Eline P Slagboom, Tim D Spector, Lisette Stolk, Aspasia Tsezou, André G Uitterlinden, Ana M Valdes, Joyce BJ van Meurs

**Affiliations:** 1 Department of Internal Medicine, Erasmus Medical Center, Rotterdam, the Netherlands; 2 The Netherlands Genomics Initiative-sponsored Netherlands Consortium for Healthy Aging (NGI-NCHA), Rotterdam/Leiden, the Netherlands; 3 Department of Molecular Epidemiology, Leiden University Medical Center, Leiden, the Netherlands; 4 Nuffield Department of Orthopaedic Surgery, Nuffield Orthopaedic Centre, Oxford, UK; 5 Laboratorio Investigacion and Rheumatology Unit, Hospital Clinico Universitario Santiago, Santiago de Compostela, Spain; 6 Twin Research and Genetic Epidemiology Unit, St. Thomas' Hospital, Kings College London, London, UK; 7 Department of Epidemiology, Erasmus Medical Center, Rotterdam, the Netherlands; 8 Department of Rheumatology and department of Clinical Epidemiology, Leiden University Medical Center, Leiden, the Netherlands; 9 University of California at San Francisco and University of California at Davis, Sacramento, USA; 10 Musculoskeletal Research Group, Newcastle University, Institute of Cellular Medicine, UK; 11 Department of Biology and Genetics, University of Thessaly, Larissa, Greece

## Abstract

**Background:**

The objective of this study was to examine the relationship between common genetic variation of the *ESR2 *gene and osteoarthritis.

**Methods:**

In the discovery study, the Rotterdam Study-I, 7 single nucleotide polymorphisms (SNPs) were genotyped and tested for association with hip (284 cases, 2772 controls), knee (665 cases, 2075 controls), and hand OA (874 cases, 2184 controls) using an additive model. In the replication stage one SNP (rs1256031) was tested in an additional 2080 hip, 1318 knee and 557 hand OA cases and 4001, 2631 and 1699 controls respectively. Fixed- and random-effects meta-analyses were performed over the complete dataset including 2364 hip, 1983 knee and 1431 hand OA cases and approximately 6000 controls.

**Results:**

The C allele of rs1256031 was associated with a 36% increased odds of hip OA in women of the Rotterdam Study-I (OR 1.36, 95% CI 1.08-1.70, p = 0.009). Haplotype analysis and analysis of knee- and hand OA did not give additional information. With the replication studies, the meta-analysis did not show a significant effect of this SNP on hip OA in the total population (OR 1.06, 95% CI 0.99-1.15, p = 0.10). Stratification according to gender did not change the results. In this study, we had 80% power to detect an odds ratio of at least 1.14 for hip OA (α = 0.05).

**Conclusion:**

This study showed that common genetic variation in the *ESR2 *gene is not likely to influence the risk of osteoarthritis with effects smaller than a 13% increase.

## Background

Epidemiological observations show sex-specific differences in the prevalence and incidence of osteoarthritis (OA) [1]: the prevalence of OA among women increases rapidly after the menopause. In addition, men have a higher prevalence of OA before the age of 50 compared to women. This has led to the hypothesis that sex hormones may be involved in the etiology of osteoarthritis [2]. Estrogen receptors α and ß are present in chondrocytes [3] and several *in vitro *and *in vivo *animal experiments showed a chondro-protective effect of estrogens [4,5]. The estrogen receptors α (*ESR1 *gene) and β (*ESR2 *gene) are nuclear proteins. Both function as ligand-regulated transcription factors and show tissue specific expression.

Previously, three studies (in total 577 cases and 1837 controls) reported an association between two Single Nucleotide Polymorphisms (SNPs) (rs2234693 and rs9340799) of the *ESR1 *gene and radiographic knee and generalized OA [6-8]. However, currently, only one small study (158 cases, 193 controls) investigated the role of variation in the *ESR2 *gene in relation to OA. A 4.5-fold increased risk of knee OA was observed in individuals carrying long alleles of the c.1092+3607(CA)n repeat polymorphism of the *ESR2 *gene [9].

In this study, we examined the relationship between common genetic variation of the *ESR2 *gene and radiographic hip-, knee- and hand osteoarthritis in a large population-based cohort study (the Rotterdam Study-I). For replication purposes, 6 additional studies were genotyped for common genetic variation in the *ESR2 *gene and a meta-analysis was performed combining all 7 studies with in total 2364 hip-, 1983 knee-, and 1431 hand OA cases and respectively 6773, 4706 and 3883 controls.

## Methods

### Selection of study populations

We searched PubMed to identify articles which could be included on this meta-analysis on common genetic variation in the *ESR2 *gene and OA. One study [9] performed an association study on common genetic variation in the ESR2 gene and OA in Caucasians, but this variant was not of interest to our study. Therefore, only novel, and therefore unbiased data, is included in this meta-analysis. Study populations with both DNA and at least hip OA data available were approached to join this meta-analysis.

### Study populations

A detailed description of all studies is described in the supplementary material (see additional file 1). In short, the discovery study is the Rotterdam Study-I, a prospective population-based cohort which comprises men and women aged 55 years and older [10]. The medical ethics committee of Erasmus University Medical School approved the study and written informed consent was obtained from each participant. The Chingford Study is another population-based longitudinal cohort, which includes 1,003 women derived from the age/sex register of a large general practice (n >11,000) in North London [11]. The Guy's St. Thomas' Trust and the Waltham Forest Trust ethics committees approved the study protocol of the Chingford Study. The Genetics osteoARthritis and Progression (GARP) study, consists of Caucasian sibling pairs and trios of Dutch origin affected by osteoarthritis at multiple sites [12]. Written informed consent was obtained from each subject involved in the GARP study as approved by the ethical committees of the Leiden University Medical Center. The Oxford TJR sample comprises subjects ascertained using the criteria of signs and symptoms of OA sufficiently severe to require joint replacement surgery in the United Kingdom [13]. Ethical approval for the Oxford collection was obtained from the Oxfordshire Clinical Research Ethics Committee, MREC 02/2/108, with each participant providing informed consent for their sample to be used in OA genetics studies. Greek OA cases are TJR patients and all are individuals of Greek origin living in the district of Thessalia in central Greece [9]. This study was approved by the ethics committee of the Larissa University Hospital and all individuals gave their informed consent. The Spanish OA cases are patients undergoing TKR/THR and were followed in the Rheumatology Unit [14]. This study was approved by the Ethical Committee for Clinical Research of Galicia and all cases and controls gave their written informed consent to participate. The Study of Osteoporotic Fractures (SOF) is a multicenter cohort study initiated in 1986 to determine risk factors for osteoporotic fractures in elderly women [15]. The SOF study was approved by the institutional review boards at each of the institutions involved. All subjects provided written informed consent at enrollment and at each clinical examination.

### Osteoarthritis

In studies with radiographic OA (ROA), radiographs were scored for the presence of ROA of the hip and knee according to the Kellgren/Lawrence (K/L) score [16]. Hip ROA was defined as at least definite JSN and a definite osteophyte and knee ROA was defined as at least 2 definite osteophytes and possible joint space narrowing. Hand OA was defined as presence of at least one definite osteophyte in 2 out of 3 hand joint groups (DIPs, PIPs, CMC1/TS) of each or both hands. Clinical studies defined hip-, hand- and/or knee- clinical OA (COA) as symptomatic OA (i.e., pain and ROA) or a TJR, which is described in the supplementary material for each study individually. In addition, one study (GARP) selected cases on the basis of both clinical and radiographic OA (CROA) at two or more joint sites among hand, spine (cervical or lumbar), knee or hip.

### Genotyping

Genomic DNA was extracted from peripheral blood leukocytes according to standard procedures. In the Rotterdam Study, we genotyped seven tagging SNPs (tSNPs): rs3020450, rs1256031, rs1256044, rs1256061, rs1109056, rs1256064 and rs4986938 (tSNP1 until tSNP7 respectively). These SNPs were selected using the program Tagger, with force include of rs1256031 and rs4986938, incorporated in Haploview. 80% of all common genetic variation in the *ESR2 *gene is covered by these 7 SNPs. In Additional file 2: **Figure S1 **the genetic variation in the *ESR2 *gene is depicted together with the D'and r^2 ^values for the 7 SNPs. We used genotype data of each of the 7 SNPs to infer frequency of the haplotype alleles using the program PHASE version 2.1[17]. Haplotypes with an estimated probability < 95% were excluded from analysis (387 individuals = 5.9%). The rs1256031 SNP was genotyped for the replication studies using a Taqman allelic discrimination assay (Chingford Study, SOF, GARP Study, Oxford Study, Greek cases and Spanish cases) (assay-on-demand service: http://www.appliedbiosystems.com) or by mass spectrometry (homogeneous Mass ARRAY system; Sequenom Inc., San Diego, CA), using standard conditions with genotypes analyzed by Genotyper 3.0 software (Sequenom Inc.)

### Quality control

The allele and genotype frequencies for rs1256064 deviated slightly from HWE proportions in the Rotterdam Study (p = 0.04), all other SNPs were in HWE proportions (data not shown). Genotyping was repeated for a random selection of subjects (5%) to check the accuracy of the genotyping. No discrepancies were detected. In addition, the allele frequency of rs1256064 is not significantly different from that reported in the CEU Hapmap population. No statistically significant deviations from HWE proportions could be detected for the rs1256031 SNP in the replication studies.

### Statistical Methods

Detailed information on the statistical methods is provided in the supplementary material (see additional file 1). In summary, odds ratios (ORs) with 95% confidence intervals (CI) were estimated with logistic regression (additive model) for all the associations between SNPs and OA phenotypes and were subsequently adjusted for gender, age and BMI (if available). The meta-analysis was performed using the program Comprehensive Meta-analysis by Biostat http://www.meta-analysis.com using fixed-effects and random-effects models. Odds ratios and 95% confidence intervals of each study were used to estimate the overall effect size for the association between SNP rs1256031 and hip OA. If the heterogeneity metric I^2 ^exceeded 25% a random-effects model (DerSimonian and Laird) was also used for the analysis, otherwise only a fixed effects model (inverse variance method) was applied. The analyses were performed on the total population of all studies and were subsequently stratified for gender to reveal, if any, gender-specific associations. A p-value ≤ 0.05 was considered statistically significant. Unless otherwise stated, SPSS version 15.0 software (SPSS INC., Chicago, USA) was used for all analyses.

## Results

### Baseline characteristics

In Table 1 the characteristics of the 7 studies are given. In total, there were 2364 hip OA cases and 6773 controls, 1983 knee OA cases and 4706 controls and 1431 hand OA cases and 3883 controls available for the meta-analysis.

**Table 1:** Baseline characteristics of all studies

	Rotterdam Study-I	Oxford Study	SOF Study	Chingford Study	GARP Study	Greek cases	Spanish cases
N (hip OA cases/controls)	284/2772	1065/727	366/1365	247/511	107/724	49/258	246/416
N (knee OA cases/controls)	665/2075	361/727	NA	302/506	148/724	258/258	249/416
N (hand OA cases/controls)	874/2184	NA	NA	99/559	244/724	NA	214/416
OA definition	ROA	COA	ROA	ROA	CROA	COA	COA
% women	58%	55%	100%	100%	70%	71%	76%
Mean age (range)	67 (55-94)	67 (55-89)	78 (72-98)	64 (54-100)	60 (43-79)	64 (18-90)	68 (55-94)
Mean BMI (range)	26 (15-59)	NA	27 (15-56)	27 (17-50)	27 (19-46)	28 (17-64)	NA

NA = not applicable, ROA = radiographic OA, COA = clinical OA, CROA = clinical and radiographic OA; SOF = Study of Osteoporotic Fractures

### Association analyses

Previously, it has been described that there is high linkage disequilibrium (LD) across the *ESR2 *region and that even between different haplotype blocks within the *ESR2 *gene there is high LD [18]. In this study we also observed high correlations (r^2 ^> 0.7) between rs1256031, rs1256044 and rs1256061 and therefore results are only presented for rs1256031 (Additional file 2: **Figure S1**). Of all 7 tSNPs tested, rs1256031 showed a significant association with hip OA in women of the Rotterdam Study-I. In Table 2, risk of OA by different genotypes of rs1256031 is given for men and women of the Rotterdam Study-I. In the Rotterdam Study-I, an allele dose effect was observed for the C-allele of rs1256031 with a 36% increased risk of hip OA in women (adjusted for age and BMI: OR1.36, 95% CI 1.08-1.70, p = 0.009). In addition, in women a trend was observed in the same direction for hand OA (OR 1.13, 95% CI 0.97-1.30, p = 0.11). No significant associations between rs1256031 and risk of OA were observed for men of the Rotterdam Study-I. Haplotype analysis did not add additional information (data not shown). There were no statistical significant associations in the Rotterdam Study-I between common genetic variation in the *ESR2 *gene and OA in a dominant or recessive model.

**Table 2:** Risk of OA according to rs1256031 (tSNP2) genotypes in the Rotterdam Study-I

		Women Rotterdam Study-I			Men Rotterdam Study-I		
***Phenotype***	***Genotype***	***Nr cases/total***^***1 ***^***(%)***	***OR (95% CI)***^***2***^	***p-value***	***Nr cases/total***^***1 ***^***(%)***	***OR (95% CI)***^***2***^	***p-value***

**Hip OA**	***TT***	43/534 (8.1)	**1.36 (1.08-1.70)**	**0.009**	34/408 (8.3)	1.01 (0.77-1.33)	0.93
	*TC*	80/861 (9.3)			58/621 (9.3)		
	*CC*	46/363 (12.7)			23/269 (8.6)		
							
**Knee OA**	***TT***	122/477 (25.6)	1.09 (0.93-1.28)	0.30	60/344 (17.4)	0.85 (0.67-1.07)	0.16
	*TC*	272/837 (32.5)			83/503 (16.5)		
	*CC*	98/346 (28.3)			30/233 (12.9)		
							
**Hand OA**	***TT***	172/510 (33.7)	1.13 (0.97-1.30)	0.11	83/418 (19.9)	0.95 (0.78-1.15)	0.58
	*TC*	300/836 (35.9)			134/644 (20.8)		
	*CC*	131/359 (36.5)			54/291 (18.6)		

^1 ^Number of cases/total number of subjects with radiographs scored for the presence of OA; ^2^additive model adjusted for age and BMI

**Figure 1:**
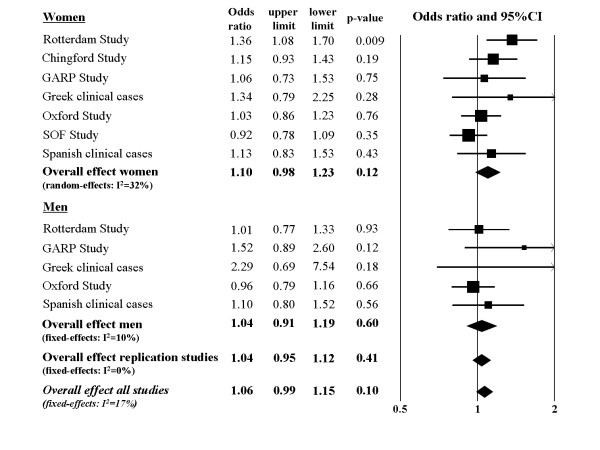
**Forest plot for the association of rs1256031 (tSNP2) with hip OA adjusted for age and BMI (allelic model)**. For the Oxford Study and Spanish cases crude odds ratio's are shown since data on BMI and/or age was not available for the majority of subjects.

Replication studies were genotyped for rs1256031 since this SNP was associated with hip OA in women of the Rotterdam Study. The allele and genotype frequencies for hip OA cases and controls in each study are presented in Table 3. The association observed with hip OA in the Rotterdam Study was not supported by the replication studies. Results of the meta-analysis for hip OA are shown as a forest plot for both men and women separately and combined in Figure 1. The replication studies together showed an OR of 1.10 (95% CI 0.98-1.23, p-value 0.12) in women. The meta-analysis showed a crude OR of 1.06 (95% CI 0.99-1.15, p-value 0.09) for hip OA, OR 1.02 (95% CI 0.94-1.10, p-value 0.62) for knee OA and OR 1.03 (95% CI 0.94-1.15, p-value 0.44 (random-effects, I^2 ^= 43%)) for hand OA. Additional adjustment for age and BMI or stratification according to gender did not essentially change the results.

**Table 3:** Allele and genotype frequencies of rs1256031 (tSNP2) for hip OA cases and controls

Study	Minor allele	MAF %		N TT		N TC		N CC	
		**Case**	**Control**	**Case**	**Control**	**Case**	**Control**	**Case**	**Control**

Rotterdam Study-I	C	48.6	45.5	77	865	138	1344	69	563
Chingford Study	C	48.6	44.7	68	159	118	247	61	105
GARP	C	48.1	45.1	28	228	55	339	24	157
Greek cases	C	53.1	43.0	12	83	22	128	15	47
Oxford Study	C	45.6	45.7	321	224	517	342	227	161
SOF	C	43.0	44.8	114	416	189	675	63	274
Spanish cases	C	46.5	44.7	73	137	117	186	56	93

MAF = minor allele frequency; N = number of subjects

## Discussion

In this study, we showed by meta-analysis of 7 studies summarizing 2364 hip OA cases and 6773 controls, 1983 knee OA cases and 4706 controls and 1431 hand OA cases and 3883 controls, that common genetic variation in the *ESR2 *gene is not likely to be associated with an increased risk of osteoarthritis.

However, we have to note that we had 80% power to detect odds ratio's of 1.14 and therefore we cannot exclude that smaller effects may exist. As only novel data was included in this meta-analysis, the risk of publication bias is eliminated by this study.

The significant association of SNP rs1256031 and hip OA in the Rotterdam Study-I was only present in women, not in men. We have previously reported that this SNP was associated with an increased risk of vertebral and fragility fractures specifically in women [18]. It was hypothesized that significant effects are not observed in men since elderly men have higher estradiol levels compared to postmenopausal women. These higher serum levels of estrogens in men may mask an impaired *ESR2 *signaling caused by the genetic variation and may also explain why we observed an association between SNP rs1256031 of the *ESR2 *gene and hip OA only in women. However, the relationship between rs1256031 and hip OA was not supported by replication studies. Previously, Patsopoulos et al. showed that claims of sex-related differences in genetic association studies are most often spurious or insufficiently documented [19]. Also in this study, where we initially did see a sex-specific association, replication studies could not corroborate this result. As the effect sizes in men and women are similar it is unlikely that interaction is present between the SNP and gender. This observation does not rule out a very subtle difference in the association between males and females and rs1256031 genotypes, but the current study is underpowered to robustly assess this.

The small case-control study by Fytili et al. (158 cases, 193 controls) reported that individuals carrying long alleles of the c.1092+3607 (CA)n repeat polymorphism of the *ESR2 *gene have a 4.5-fold increased risk of knee OA. Since this repeat polymorphism was not studied in this meta-analysis and it is not known whether this repeat is in LD with rs1256031 we cannot conclude that we did or did not replicate the finding of the case-control study of Fytili and co-workers [9].

At this moment, genome-wide association studies (GWAS) are state-of-art studies to indentify novel genetic loci involved in complex diseases like OA. In the genome-wide association studies published to date, common genetic variation in the *ESR2 *gene has not been found associated with OA [20-23]. In addition, GWAS on bone-related traits like bone mineral density (BMD) did also not observe any genome-wide significant associations between common genetic variation in the *ESR2 *gene and BMD [24-26].

## Conclusion

It is not likely that there is an association between common genetic variation in the *ESR2 *gene and hand-, hip- or knee OA although associations with very small effect sizes can not be excluded.

## Competing interests

The authors declare that they have no competing interests.

## Authors' contributions

Conception and design: HK, JM, AU, LS, FR, HP. Acquisition of data: AH, HP, AU, IM, AC, AG, JL, DH, MK, NL, MN, ES, TS, AT, AV. Analysis and interpretation of data: HK, IM, NL. Drafting the manuscript: HK, JM. Revising of manuscript: all authors. Final approval of the manuscript: all authors

## Pre-publication history

The pre-publication history for this paper can be accessed here:

http://www.biomedcentral.com/1471-2350/11/164/prepub

## Supplementary Material

Additional file 1 **Description of study populations and statistical methods**. Click here for file

Additional file 2 **Figure S1: The *ESR2 *gene Linkage Disequilibrium (D') and correlation (R^2^) between the tagging SNPs**. Each box in the table represents D'or R^2 ^for the two SNPs indicated. Click here for file
