# Supplementary material for: Common genetic variation in the Estrogen Receptor Beta (ESR2) gene and osteoarthritis: results of a meta-analysis
Source: BMC Med Genet. 2010 Nov 16;11:164. doi: 10.1186/1471-2350-11-164 (PMC2997092; doi:10.1186/1471-2350-11-164)
Supplement: Additional file 1 — Description of study populations and statistical methods. [file 1471-2350-11-164-S1.DOC]

# Supplementary material:

# *Study populations:*

The Rotterdam Study-I: the Rotterdam Study is a prospective population-based study on determinants of chronic disabling diseases. The study population comprises men and women aged 55 years and older. The rationale and study design have been described previously [1]. Subjects were scored for the presence of OA using standardized radiographs of the hip, knee and hand. In total, there were 874 hand OA cases and 2184 controls, 665 knee OA cases and 2075 controls and 284 hip OA cases and 2772 controls.

The Chingford Study: is a prospective population-based longitudinal cohort, which includes women derived from the age/sex register of a large general practice in North London [2, 3]. The study design and rationale have been described elsewhere in detail [4]. After study procedures were explained to participants, written informed consent was given by each participant. In total, there were 99 hand OA cases and 559 controls, 302 knee OA cases and 506 controls and 247 hip OA cases and 511 controls.

The Genetics OsteoArthritis and Progression (GARP) study from Leiden, the Netherlands, consisted of 192 sibling pairs concordant for clinical and radiographically (K/L score) confirmed OA at two or more joint sites among hand, spine (cervical or lumbar), knee or hip, random controls (N=720) were partners of the offspring of the Leiden longevity study [9]. To comply with the discovery sample OA phenotypes for knee, hip and hand OA used were based on radiographic signs of OA. In total, there were 107 hip-, 148 knee-, and 244 hand OA cases and 724 controls.

Greek TJR cases: the individuals included in the study were of Greek origin living in the district of Thessalia in central Greece [6]. All of them had undergone a TKR/THR, meaning that all of them suffered from severe knee or hip OA, which is defined by a K/L grade >=2. None of the patients had evidence of arthritis due to another disease. All the controls had a K/L score of 0 and had undergone treatment for injuries or fractures. Patients with rheumatoid arthritis and other autoimmune diseases as well achondrodysplasias, infection-induced OA, and posttraumatic OA were not included in the study. In total, 49 hip OA cases and 211 controls, and 258 knee OA cases and 258 controls were available.

The Oxford Study: subjects were ascertained using the criteria of signs and symptoms of OA sufficiently severe to require joint replacement surgery in the United Kingdom [5]. The radiographic stage of the disease was a K/L grade >=2 in all cases. In addition, no cases suggestive of a skeletal dysplasia or developmental dysplasia were included. The controls comprised individuals with no signs or symptoms of arthritis or joint disease (pain, swelling, tenderness or restriction of movement). Only for a subset of the samples data on age was available. In total, there were 361 knee- and 1065 hip OA cases and 727 controls.

Study of Osteoporotic Fractures (SOF) is a multicenter cohort study initiated in 1986 to determine risk factors for osteoporotic fractures in elderly women [10]. Participants were all aged > 65 years at baseline and were recruited from population-based listings at 4 clinical centers in the US: Baltimore, MD; Minneapolis, MN; Monongahela Valley, PA (near Pittsburgh); and Portland, OR. Exclusion criteria for the parent study, the SOF, included bilateral hip replacement and an inability to walk unassisted. In total, there were 366 hip-OA cases and 1365 controls available for association analysis.

Spanish TJR and hand-OA cases: patients were selected from consecutive patients, aged 55-75 years of age at time of the surgery, undergoing THR/TKR and patients complaining of hand OA that were followed in the Rheumatology Unit [7]. All patients were included if a rheumatologist considered them to suffer from severe primary OA. Exclusion criteria were inflammatory, infectious, traumatic or congenital joint pathology and lesions due to crystal deposition or osteonecrosis. Patients with hand OA were required to fulfill the ACR criteria [8]. Controls were recruited among subjects older than 55 years of age undergoing preoperative work-up for elective surgeries other than joint surgery and who did not show clinical manifestations of OA. In total, there were 246 hip OA-, 249 knee OA-, 214 hand OA cases and 416 controls.

*Statistical Methods:* for the individual SNPs, allele frequencies were estimated by allele counting and Hardy-Weinberg Equilibrium (HWE) was tested using Haploview. Differences in baseline characteristics were evaluated by analysis of co-variance (ANCOVA). Odds ratios (ORs) with 95% confidence intervals (CI) were estimated with logistic regression for all the OA outcomes and were subsequently adjusted for gender, age and BMI. In the Rotterdam Study-I, the discovery study, additive models as well as dominant and recessive models were tested. In the Oxford Study and for the Spanish cases it was not possible to adjust for age and BMI since only part of the subjects had data available for these covariates. For the GARP study p-values were adjusted for family relationships by using robust standard error analyses, using Stata SE8 software (Stata corporation, College Station, TX) [5].

**References:**

[1] Hofman A, Breteler MM, van Duijn CM, Janssen HL, Krestin GP, Kuipers EJ, Stricker BH, Tiemeier H, Uitterlinden AG, Vingerling JR, Witteman JC. **The Rotterdam Study: 2010 objectives and design update**. *Eur J Epidemiol* 2009, **24**:553-72.

[2] Hart DJ, Spector TD. **The relationship of obesity, fat distribution and osteoarthritis in women in the general population: the Chingford Study**. *J Rheumatol* 1993, **20**:331-5.

[3] Hart DJ, Spector TD. **Cigarette smoking and risk of osteoarthritis in women in the general population: the Chingford study.** *Ann Rheum Dis* 1993, **52**:93-6.

[4] Hart DJ, Mootoosamy I, Doyle DV, Spector TD. T**he relationship between osteoarthritis and osteoporosis in the general population: the Chingford Study**. *Ann Rheum Dis* 1994, **53**:158-62.

[5] Chapman K, Takahashi A, Meulenbelt I, Watson C, Rodriguez-Lopez J, Egli R, Tsezou A, Malizos KN, Kloppenburg M, Shi D, Southam L, van der Breggen R, Donn R, Qin J, Doherty M, Slagboom PE, Wallis G, Kamatani N, Jiang Q, Gonzalez A, Loughlin J, Ikegawa S. **A meta-analysis of European and Asian cohorts reveals a global role of a functional SNP in the 5' UTR of GDF5 with osteoarthritis susceptibility.** *Hum Mol Genet* 2008, **17**:1497-504.

[6] Fytili P, Giannatou E, Papanikolaou V, Stripeli F, Karachalios T, Malizos K, Tsezou A. **Association of repeat polymorphisms in the estrogen receptors alpha, beta, and androgen receptor genes with knee osteoarthritis.** *Clin Genet* 2005, **68**:268-77.

[7] Rodriguez-Lopez J, Pombo-Suarez M, Liz M, Gomez-Reino JJ, Gonzalez A. **Lack of association of a variable number of aspartic acid residues in the asporin gene with osteoarthritis susceptibility: case-control studies in Spanish Caucasians.** *Arthritis Res Ther* 2006, **8**:R55.

[8] Altman RD, Fries JF, Bloch DA, Carstens J, Cooke TD, Genant H, Gofton P, Groth H, McShane DJ, Murphy WA. **Radiographic assessment of progression in osteoarthritis.** *Arthritis Rheum* 1987, **30**:1214-25.

[9] Heijmans BT, Beekman M, Houwing-Duistermaat JJ, Cobain MR, Powell J, Blauw GJ, van der Ouderaa F, Westendorp RG, Slagboom PE. **Lipoprotein particle profiles mark familial and sporadic human longevity.** *PLoS Med* 2006, 3:e495.

[10] Cummings SR, Nevitt MC, Browner WS, Stone K, Fox KM, Ensrud KE, Cauley J, Black D, Vogt TM. **Risk factors for hip fracture in white women. Study of Osteoporotic Fractures Research Group.** N *Engl J Med* 1995, **332**:767-73.
