# Supplementary figures and images for: Common genetic variation in the Estrogen Receptor Beta (ESR2) gene and osteoarthritis: results of a meta-analysis
Source: BMC Med Genet. 2010 Nov 16;11:164. doi: 10.1186/1471-2350-11-164 (PMC2997092; doi:10.1186/1471-2350-11-164)

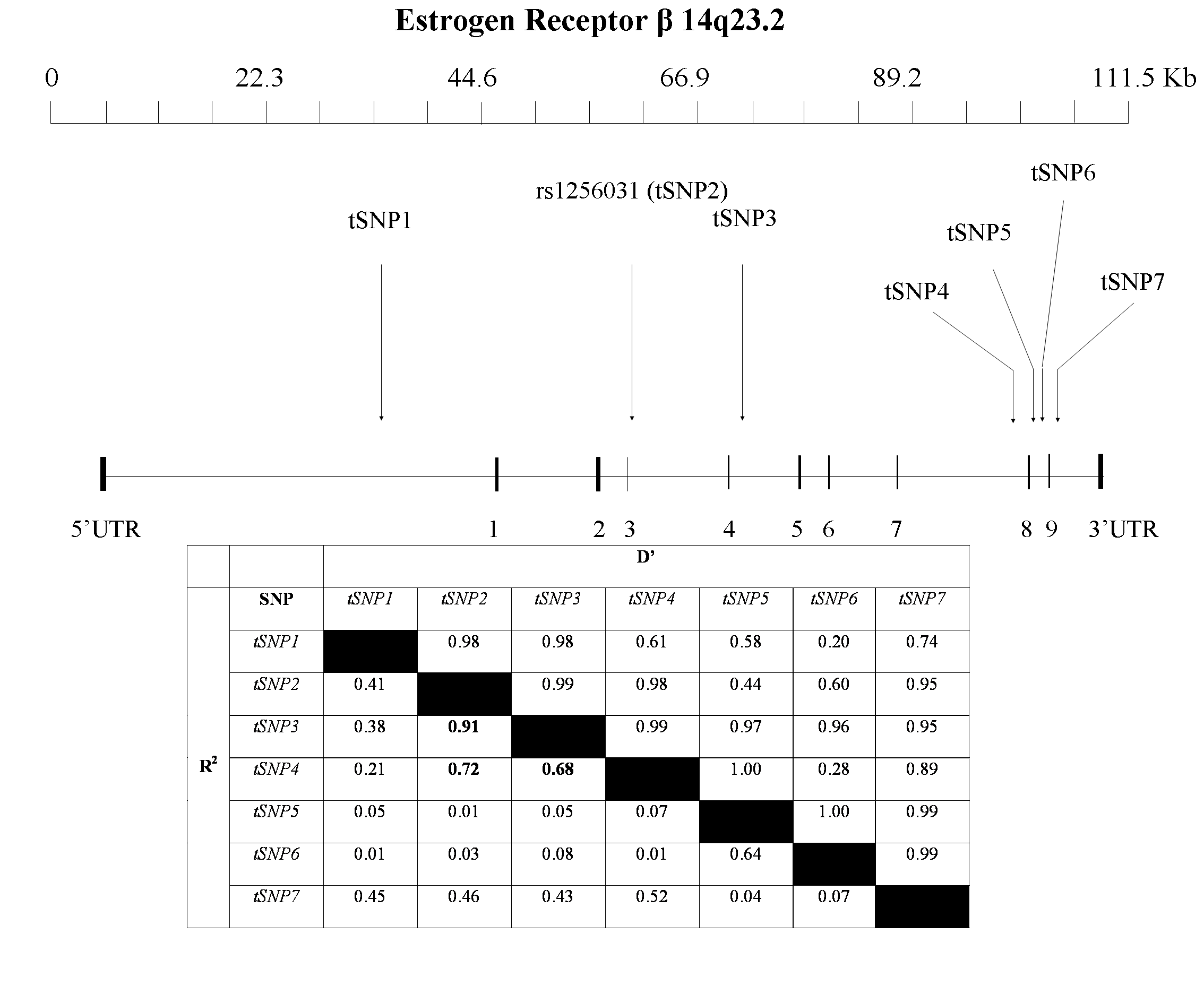

Supplement: Additional file 2 — Figure S1: The ESR2 gene Linkage Disequilibrium (D') and correlation (R2) between the tagging SNPs. Each box in the table represents D'or R2 for the two SNPs indicated. [file 1471-2350-11-164-S2.TIFF]
